# Rehabilitation of a Pregnant Patient With Guillain-Barré Syndrome: A Physiotherapy Perspective

**DOI:** 10.7759/cureus.88340

**Published:** 2025-07-19

**Authors:** Ruchita Killedar, Gauri Wakde, Sampada Kulkarni, Apurva Jadhav, Swati Bhise

**Affiliations:** 1 Cardiovascular and Respiratory Physiotherapy, School of Physiotherapy, Bharati Vidyapeeth (Deemed to be University), Pune, IND; 2 Anaesthesiology, Bharati Hospital and Research Centre, Pune, IND; 3 Neurophysiotherapy, School of Physiotherapy, Bharati Vidyapeeth (Deemed to be University), Pune, IND

**Keywords:** critical care physiotherapy, gbs in pregnancy, guillain-barre syndrome, physiotherapy, rehabilitation

## Abstract

Guillain-Barré syndrome (GBS) is an acute autoimmune polyneuropathy that leads to muscle weakness and substantial respiratory dysfunction. This case elaborates the detailed physiotherapeutic approach for the management of muscular weakness and respiratory dysfunction in a 39-year-old pregnant woman diagnosed with GBS in her second trimester. The patient received vigorous physiotherapy for eight weeks, during which she was on ventilatory support for 40 days. Through specialized physiotherapeutic approaches tailored to her neurological condition and pregnancy status, she demonstrated significant improvements in mobility, respiratory function, and muscle control.

## Introduction

Guillain-Barré syndrome (GBS) is an autoimmune polyneuropathy affecting the peripheral nerves, characterized by flaccid paralysis of all four limbs. The global incidence of GBS ranges from 1.2 to 2.3 cases per 100,000 persons [1,2]. GBS usually presents with a preceding infection, and the onset is typically sudden. Depending on the clinical presentation, GBS can be classified into three types: pure motor, pure sensory, or motor-sensory. It can also be categorized into acute inflammatory demyelinating polyneuropathy (AIDP), acute motor axonal neuropathy (AMAN), acute motor sensory axonal neuropathy (AMSAN), Miller Fisher syndrome (MFS), and a pure sensory type of GBS [3]. The diagnosis of GBS is primarily evident through clinical symptoms characterized by the presence of acute flaccid paralysis, accompanied by the absence or reduction of deep tendon reflexes. The confirmed diagnosis of GBS is typically concluded through electrophysiological studies, including nerve conduction studies and electromyography (EMG), as well as cerebrospinal fluid (CSF) analysis [4].

GBS can occur in any trimester of pregnancy or in the postpartum period. The estimated incidence of GBS in pregnancy is 1.2 to 1.9 cases per 100,000 people annually [5]. The diagnosis of GBS may also be delayed in pregnancy since the symptoms of GBS are likely to mimic those of pregnancy. A diagnosis of GBS typically presents with muscle weakness, general malaise, tingling in the fingers, and difficulty in breathing. The nerve conduction velocity (NCV) studies and CSF analysis are the gold standard methods for confirming the diagnosis of GBS. The aim of physiotherapy interventions is to accelerate recovery, reduce severity, shorten hospital stay, alleviate financial burden, and improve quality of life [6].

This case represents a 39-year-old pregnant female with complaints of low-grade intermittent fever, progressive tingling sensation, and weakness in both legs with a previous history of GBS. The management included intensive care and rigorous physiotherapy. This study aimed to emphasize the rarity of GBS in pregnant women, highlighting the role of physiotherapy along with intensive care management.

## Case presentation

Discharge summary of previous hospitalization

A 39-year-old female patient at 22 weeks of gestation presented with a history of low-grade intermittent fever, progressive tingling sensation, and weakness in both legs on September 15, 2024. The next day, the patient experienced progressive weakness in both upper and lower limbs, which was followed by difficulty in walking and slurred speech. She consulted her obstetrician on September 17, 2024, who referred her further to a neurologist due to the atypical nature of her symptoms. She was hospitalized on the same day, and she progressively worsened on day two of admission, that is, September 18, 2024. Her chest X-ray showed left-sided lung collapse. She developed respiratory distress and thus required intubation and ventilatory support. Emergency bronchoscopy was performed. A nerve conduction study performed on September 18, 2024, showed severe generalized motor axonal neuropathy in both upper and lower limbs.

During this hospitalization, she underwent lumbar puncture, bronchoscopy, tracheostomy, and bronchoscopy with bronchoalveolar lavage. She reported a past history of GBS at the age of 16 (treatment details not available). The patient underwent a brain CT scan and lumbar puncture (CSF examination), leading to a diagnosis of GBS. She received intravenous immunoglobulin therapy the same day. The patient reported progressive dysesthesia affecting her lower and upper extremities and tongue, resulting in slurred speech.

Normal fetal development was confirmed via ultrasound. At the time of discharge, the patient was on anticoagulant (injection Clexane), proton pump inhibitor (Tab PAN), hormonal support (Tab Dydrogesterone), laxative (Liq Duphalac), calcium and vitamin D supplement (Tab Shelcal), Formonide Respule (inhaled corticosteroid and bronchodilator), and nebulization of Musinac.

Transfer to our facility: status at admission

On October 25, 2024, the patient was transferred to our facility for further management under the Neuro-medicine and Critical Care Medicine departments. She was admitted to the intensive care unit (ICU). Upon transfer, the patient was conscious and oriented to time and place, with a tracheostomy tube in situ. Her respiratory rate was 40 breaths per minute, blood pressure was 180/100 mmHg, pulse rate was 125 beats per minute, and oxygen saturation was 84%. There was moderate peripheral edema in the bilateral lower limbs. The overall neuropathy limitation scale score was 12/12 (complete disability). Touch, pain, and temperature sensations were diminished all over. Ultrasound confirmed normal fetal status. The patient was diagnosed with GBS at 24 weeks of gestation, acute viral respiratory infection (AVRI), and iron deficiency anemia. The patient spent 40 days in the critical care unit and 15 days in the step-down unit and was subsequently discharged.

Manual muscle testing (MMT) revealed severe muscle weakness, as indicated by a Medical Research Council (MRC) scale score of zero, along with sensory deficits in both the bilateral upper and lower limbs. Reflexes were altered in both upper and lower extremities. The patient received extensive physiotherapy management over the course of 55 days (eight weeks) in our facility.

Obstetric outcome

An emergency lower-segment cesarean section (LSCS) was performed on November 5, 2024, due to preterm labor. The baby required immediate intubation and resuscitation, with APGAR scores of 1 and 2 at one and five minutes, respectively. The newborn was transferred to the neonatal intensive care unit (NICU) with respiratory acidosis. The baby was managed for congenital pneumonia with surfactant therapy and mechanical ventilation. Deep cyanosis and hypotension were treated with inotropes. Phototherapy was given for hyperbilirubinemia. The baby was also managed for hypoxic-ischemic encephalopathy with resolving bilateral germinal matrix hemorrhage. At two months, the baby was maintaining well without any oxygen support and was discharged from the hospital.

Physiotherapy management in the course of 55 days

Respiratory Rehabilitation

Initially, the patient was on pressure-controlled ventilation (PCV) mode with FiO_2_ of 80% for approximately two weeks, followed by synchronized intermittent mandatory ventilation (SIMV) mode with FiO_2_ of 60% between weeks two and three. During weeks three to four, a trial was taken for CPAP (continuous positive airway pressure) mode, which later progressed to BiPAP (bilevel positive airway pressure) mode by week six. By week eight, the patient was able to maintain saturation with O_2_ support of 2 L/minute via tracheostomy tube and intermittent BiPAP.

The chest physiotherapy maneuver primarily involved postural drainage, coupled with mechanical vibrations, followed by suctioning. Suctioning was done as and when required. Proprioceptive neuromuscular facilitation (PNF) techniques, such as anterior basal stretch lift, intercostal stretch, and vertebral pressure, were administered for 8-10 repetitions until a visible increase in chest expansion was observed [7]. Diaphragmatic breathing exercises were encouraged from day one and showed significant improvement in chest expansion.

During this period, the patient underwent a lung recruitment maneuver, in which a temporary high PEEP (positive end-expiratory pressure) was applied to maintain alveoli patency, thereby opening collateral channels [8,9]. A change of position every two hours was given to facilitate the opening of collateral channels and also to improve the ventilation-perfusion mismatch. This change in position also helped prevent bedsores and skin irritation resulting from prolonged bedridden status. The chest physiotherapy sessions were delivered three times a day, with each session lasting 30-40 minutes. Incentive spirometry was started via tracheostomy tube in the second week [10].

**Figure 1:**
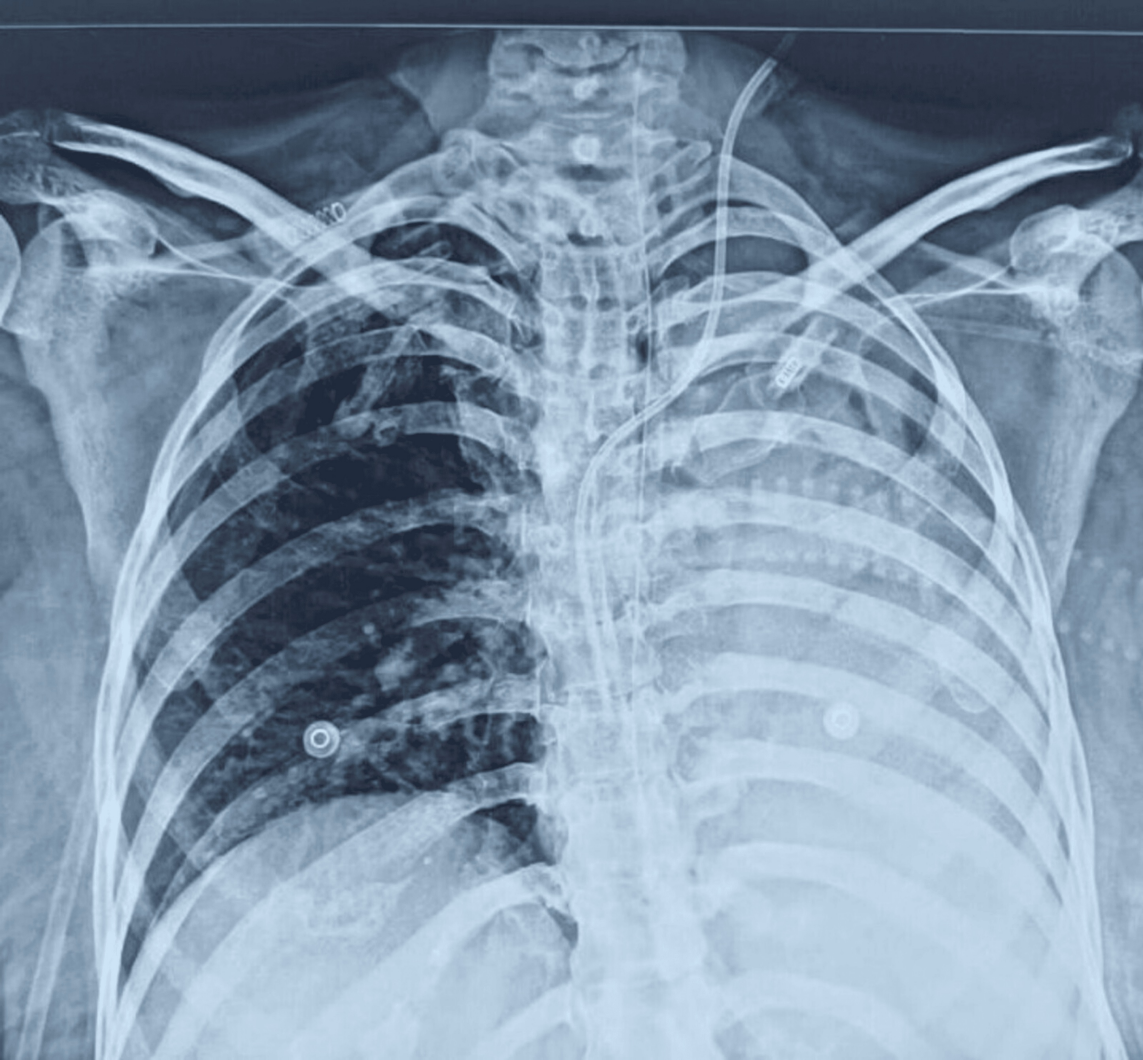
Chest radiograph taken at two weeks from transfer to our facility, where the patient had developed left-sided ventilator-acquired pneumonia. This resolved in five days.

**Figure 2:**
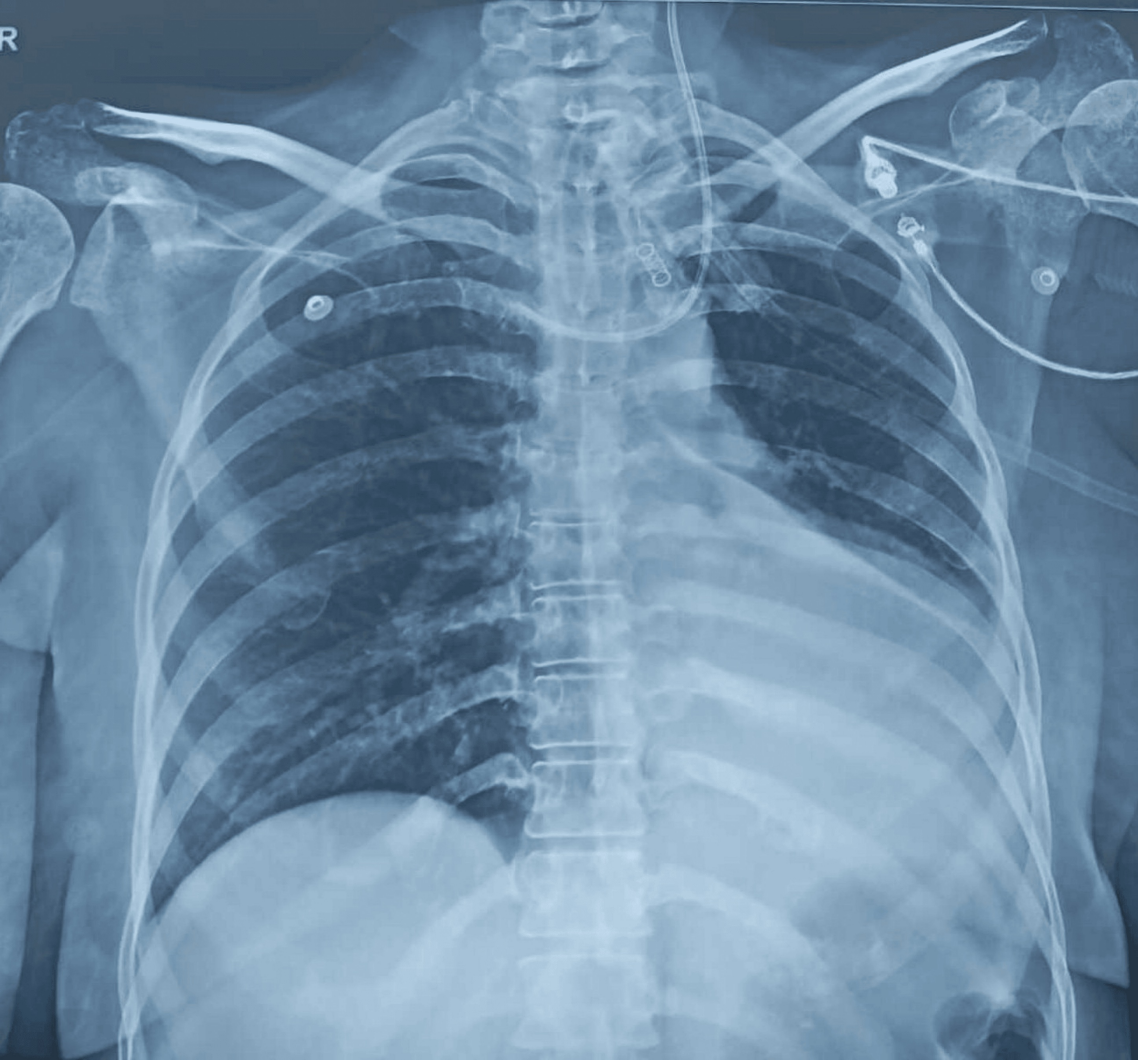
A chest radiograph was taken after the resolution of pneumonia, five days later. Chest physiotherapy was continued in conjunction with medical management.

Initially, the patient's efforts were minimal, which eventually resulted in a 600 cc volume of air in three weeks, progressing to 900 cc by the eighth week. During the course, the patient developed ventilator-associated pneumonia twice, once in the left lower lobe and once in the right lower lobe. Both times, pneumonia resolved within five days, and chest physiotherapy was continued throughout the course of the infection, along with medical management (Figures 1, 2).

Neurological rehabilitation

Weeks 1-2

During the first week of intervention, passive range of motion (PROM) exercises were administered to all major joints in the pain-free arc of movement, which helped to preserve joint mobility and prevent musculoskeletal complications. An ankle-foot orthosis (AFO) was also incorporated in the protocol to maintain the ankle in a neutral position to prevent plantar flexor contractures. This helped avoid the risk of long-term deformities and improve functional outcomes [11-13].

**Figure 3:**
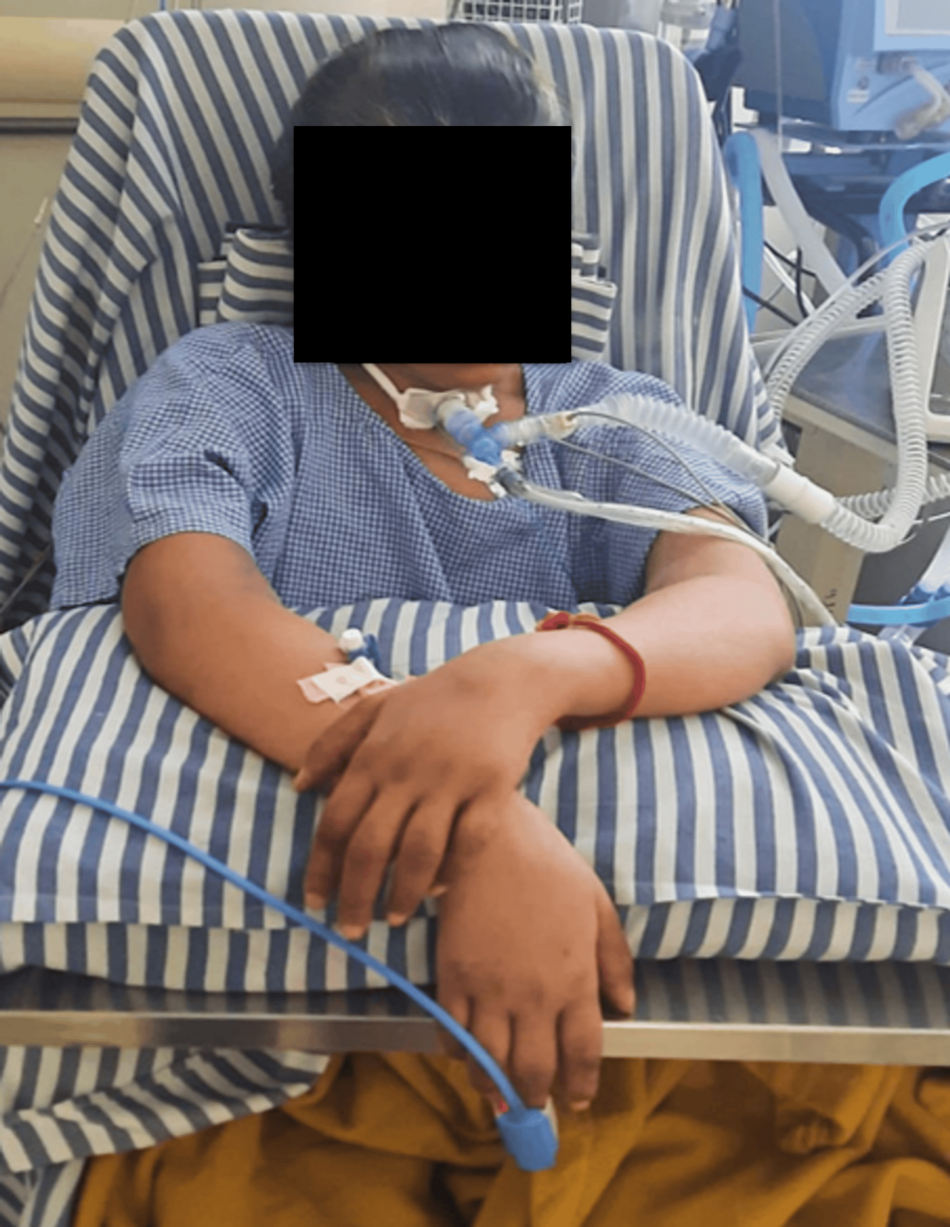
Sitting in bed with assistance.

The neurodevelopmental therapy (NDT) approach [14] was an integral part of rehab, which helped to promote neuromotor development. In this, assisted rolling techniques were used with wedges to facilitate positioning. These strategies are designed to stimulate gross motor skills and enhance postural control. A supported sitting posture was encouraged early in the rehabilitation process, aided by adaptive seating devices (Figure 3). This not only promoted early mobilization but also laid the foundation for postural alignment and weight-bearing tolerance. Facilitatory techniques, including quick icing for large muscle groups such as the quadriceps and biceps, joint approximation, quick stretching, and PNF methods, were employed in conjunction with PROM to enhance neuromuscular responsiveness and sensorimotor integration [15,16].

Caregiver education was always prioritized during the rehabilitation process, with a focus on sensory integration therapy. We included frequent sensory input using textured materials to increase awareness and tolerance to various sensory stimuli. A customized sensory stimulation kit was developed, specifically tailored to the patient's needs, consisting of a range of tactile inputs, including sand, grains, clay, and other textural elements. These were administered regularly to facilitate and stimulate multisensory engagement. This was well executed by the caregiver.

Weeks 2-3

There was a gradual improvement in tone in the week after the emergency LSCS. In this phase, her psychological well-being was drastically affected due to the critical condition of her newborn, who was admitted to the NICU. Considering the significant emotional toll, psychological counseling and supportive therapies were initiated by trained professionals, which played a key role in helping her cope with anxiety and emotional distress while continuing her physical recovery.

Active mobilization was introduced gradually as there was a notable improvement in muscle tone. To facilitate motor control, neurodevelopmental positions, such as supine-to-sit transitions and rolling techniques, were incorporated alongside range of motion exercises with appropriate support to protect the LSCS incision site during all exercise sessions. PNF techniques, such as rhythmic initiation, D1 flexion-extension patterns for the upper limbs, D2 flexion-extension patterns for the lower limbs, and rhythmic stabilization for trunk muscles, were applied to reinforce muscle activity and promote coordinated motor unit activation. Passive contract-relax techniques were also introduced during this phase [17,18].

Wheelchair ambulation was initiated during this period. The patient was now taken out of the critical care unit once daily under supervision. Neuromuscular electrical stimulation (NEMS) therapy was introduced to support neuromuscular activation. This was administered once daily for 20 minutes, with one limb targeted per session. This rehabilitation approach facilitated both physical and psychological progress during a critical stage in the patient's recovery [19].

Weeks 3-4

Vertical posture training using a tilt table was initiated on alternate days, which helped promote weight-bearing and stimulate joint proprioceptors. This intervention aimed to counteract the pernicious effects of prolonged immobilization, such as muscle atrophy and orthostatic hypotension, while gradually enhancing the patient’s tolerance to an upright, standing position. The sessions were scheduled on alternate days to prevent fatigue and minimize the risk of hypokalemia, which can be a concern in critically ill patients [20].

**Figure 4:**
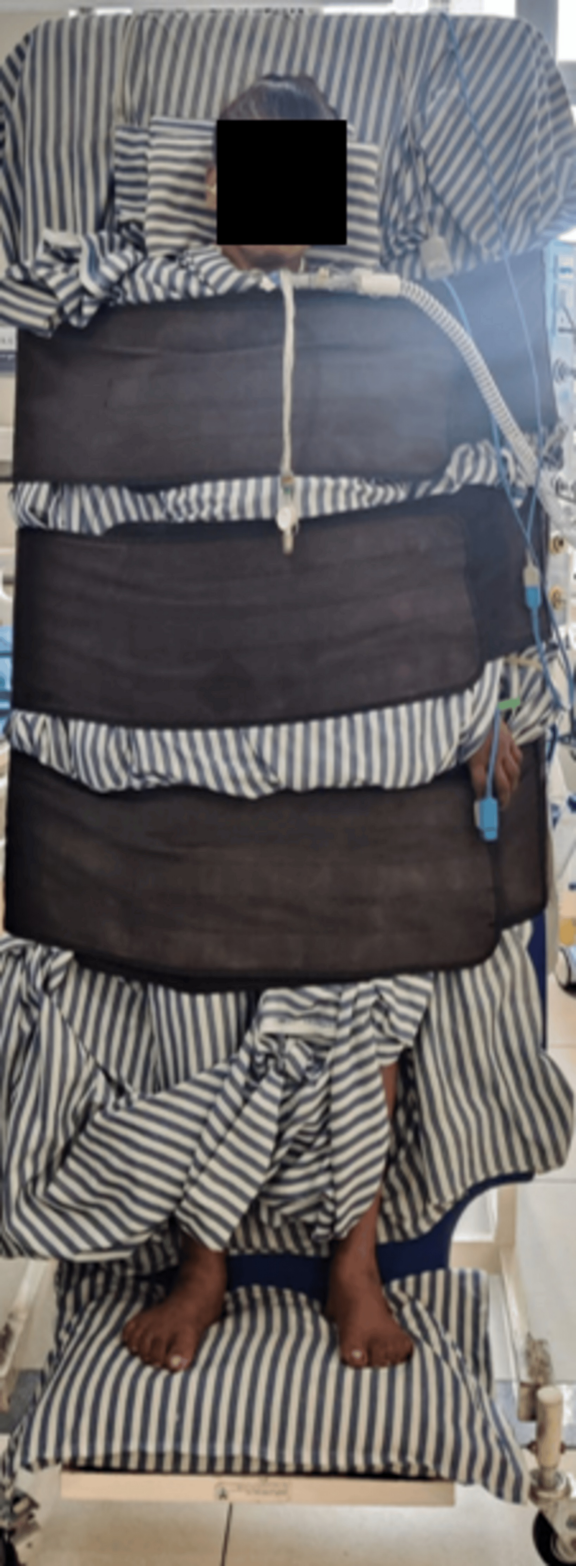
Verticalization using a tilt table.

A non-fatiguing strengthening program was initiated to improve muscle recruitment and functional strength. This included active-assisted range of motion (AAROM) exercises and isometric contraction training for larger muscle groups, specifically performed at the mid-arc of movement, prioritizing both safety and efficacy. Fixation or limb-holding exercises were incorporated to improve static muscle endurance and joint stability. PNF techniques, particularly rhythmic stabilization for both upper and lower extremities, were continued as part of the therapeutic regimen to enhance postural control and neuromuscular coordination (Figure 4) [21].

Weeks 4-8

In the subsequent course of rehabilitation, interventions were focused on enhancing postural control and developing fall-coping strategies. To achieve this, multidirectional reach training, weight-shifting exercises in all directions, and equilibrium reaction training in the sitting position were incorporated. These activities aimed to improve balance, trunk stability, and dynamic postural adjustments, which are essential for achieving upright mobility. An attempt was made to initiate treadmill training with partial body weight support; however, it was not successful due to the patient's poor balance and insufficient overall muscle tone.

To continue promoting neuromuscular engagement, neurodevelopmental positioning strategies were applied using assistive devices to facilitate heavy joint compression throughout the limbs, thereby aiding in the improvement of muscle tone. Functional positions such as prone-on-elbows and quadruped were introduced with assistance to stimulate weight-bearing and proximal joint stability. Progressive resisted strengthening exercises were advanced using active range of motion in a gravity-eliminating plane. Specific PNF patterns were also incorporated, such as chopping patterns for trunk strengthening and kicking patterns for lower extremities, aimed at restoring coordinated motor function.

Weight-bearing in the lower limbs was encouraged through assisted standing, utilizing supportive aids like Pedi wraps to maintain knee extension and prevent buckling. However, a significant challenge throughout the first four weeks of rehabilitation was the presence of intermittent episodes of bradycardia. These brief but recurrent drops in heart rate would resolve spontaneously, but they hindered consistent rehabilitation progress [22,23].

## Discussion

Physiotherapy plays a pivotal role in the rehabilitation of patients with GBS, with studies reporting complete recovery in 58% of patients and an overall satisfaction rate of 87% [4]. When GBS occurs during pregnancy, it presents unique challenges, requiring a careful balance between maternal and fetal well-being while addressing profound neuromuscular deficits. In such cases, physiotherapy serves as a cornerstone of multidisciplinary management, aiming to restore functional independence, enhance mobility, and improve overall quality of life.

In this case, the patient was ventilator-dependent for 40 days, and physiotherapy interventions were initiated early to support respiration and prevent complications. Techniques such as postural drainage, chest vibrations, and tracheostomy suctioning were administered routinely. These measures aided mucociliary clearance, prevented microatelectasis, and reduced the risk of respiratory infections. The patient also experienced repeated episodes of pneumonia, for which chest physiotherapy played a critical role in improving the ventilation-perfusion mismatch and facilitating recovery. To support ventilatory management, additional strategies such as frequent position changes, breath synchronization techniques, and lung recruitment maneuvers using higher PEEP were employed. These interventions helped open collapsed alveoli, enhance thoracic mobility, and improve gas exchange. Ventilator hyperinflation was also utilized to mobilize secretions, promote lung expansion, and assist in the weaning process [8-10].

A stepwise and individualized physiotherapy plan was central to the patient's recovery. Initially, PROM exercises were used to maintain joint integrity and prevent contractures. As the patient's condition stabilized, the focus shifted to active-assisted movements and wheelchair transfer training. These early mobility efforts helped build cardiovascular endurance and contributed to improved psychological well-being. The introduction of tilt table training marked a key milestone in the rehabilitation process. By promoting orthostatic adaptation and initiating weight-bearing through the lower limbs, the tilt table helped reduce postural hypotension and facilitated early functional engagement. Subsequently, strengthening exercises targeting anti-gravity muscle groups were introduced. These progressive resistance exercises helped reduce pain and rebuild muscle strength, with particular emphasis on core stability and lower limb control. Functional re-education, including sit-to-stand and transfer training, further supported the patient's transition toward independence. These interventions not only improved strength and endurance but also contributed to the prevention of complications and enhanced mental resilience [13,14].

Neuromuscular flaccidity was addressed through PROM, joint compressions, and weight-bearing exercises, which collectively helped modulate tone and improve motor control. To prevent contractures, passive contract-relax stretching techniques were applied, facilitating muscle elongation. Additionally, anti-foot-drop splints were provided early in the course of care to maintain proper limb positioning. NMES (neuromuscular and muscular electrical stimulation) was also incorporated to enhance neuromuscular activation [15].

Considering the outcome of the treatment, the patient demonstrated significant functional improvement, as evidenced by a reduction in the Hughes Functional Grading Scale score from 5 to 3, an improvement in the Overall Neuropathy Limitations Scale (ONLS) from 12 to 7, and a substantial increase in the Functional Independence Measure (FIM) score from 12 to 49 (Table 1). These improvements were supported by noticeable gains in muscle tone (Table 2), with reflexes progressing from absent (0) to 1+ (Table 3) and enhanced muscle strength across various muscle groups as assessed by MMT (Table 4). Collectively, these neuromuscular improvements contributed meaningfully to the patient's overall recovery and increased level of independence. The administration of TENS (transcutaneous electrical nerve stimulation) therapy may have contributed to a reduction in pain levels, as reflected by improvements in the Numerical Pain Rating Scale (NPRS) (Table 1).

**Table 1:** Comparative summary of functional scales between the day of admission and discharge. NPRS: Numerical Pain Rating Scale; ONLS: Overall Neuropathy Limitations Scale

Outcome Measures	At Hospitalization	At Discharge
Hughes scale	5	3
NPRS scale	9	4
Functional independence scale	18	49
ONLS	12	7

**Table 2:** Comparative summary of muscle tone between the day of hospitalization and discharge.

Extremity	At Hospitalization	At Discharge
Upper Extremity muscles	Flaccid	Hypotonia
Lower Extremity muscles	Flaccid	Hypotonia

**Table 3:** Comparative summary of reflexes between the day of hospitalization and discharge.

Types of Reflex	At Hospitalization	At Discharge
Superficial reflexes		
Planter	Absent	Present
Abdominal	Absent	Present
Deep tendon reflexes		
Biceps	0	1+
Triceps	0	1+
Knee jerk	0	1+
Ankle jerk	0	1+

**Table 4:** Comparative summary of manual muscle testing between the day of hospitalization and discharge.

Muscle Group	At Hospitalization	At Discharge
Shoulder flexors	0	2
Shoulder extensors	0	1
Shoulder abductors	0	1
Elbow flexors	0	2
Elbow extensors	0	1
Hip extensors	0	2
Hip flexors	0	1
Ankle dorsiflexors	0	0
Ankle plantarflexors	0	0
Knee flexors	0	1
Knee extensors	0	2

The patient's ability to stand with support is a promising outcome that underscores the effectiveness of a comprehensive rehabilitation program. Looking ahead, therapy will continue to emphasize respiratory health through regular breathing exercises and the use of an incentive spirometer. Weight-shifting drills, balance training, and proprioceptive activities will be introduced to improve postural control and coordination. Gait training, beginning with assistive devices and progressing to unassisted ambulation, will be tailored according to the patient's functional status. Ongoing strengthening exercises and encouragement to resume daily activities will aim to restore independence and promote reintegration into the community.

## Conclusions

The physiotherapeutic management of a pregnant woman diagnosed with GBS demands a comprehensive and individualized approach, particularly during the acute phase of the illness. In this case report, we highlighted the critical role of physiotherapy in addressing the dual challenge of managing a neuromuscular disorder within the context of pregnancy. Our intervention primarily focused on acute care rehabilitation strategies aimed at preventing respiratory complications, maintaining joint range of motion, and promoting safe mobilization. Special consideration was given to the patient's altered biomechanics and the safety of both mother and fetus. A systematic and multidisciplinary approach was employed, emphasizing respiratory care through breathing exercises and airway clearance techniques, as well as progressive muscle strengthening and early mobility training. These interventions were introduced cautiously and sequentially, based on the patient's tolerance and medical stability. The collaborative efforts of the healthcare team ensured close monitoring and timely adjustments to the rehabilitation program. The positive recovery trajectory observed in this patient underscores the importance of early physiotherapy in acute care settings. Continued physiotherapeutic input throughout the recovery period significantly contributes to improved functional outcomes, greater independence in daily activities, and ultimately a better quality of life, even in the presence of complex clinical conditions, such as GBS during pregnancy.
